# A Fuzzy Controller for Lower Limb Exoskeletons during Sit-to-Stand and Stand-to-Sit Movement Using Wearable Sensors

**DOI:** 10.3390/s140304342

**Published:** 2014-03-04

**Authors:** Sharif Muhammad Taslim Reza, Norhafizan Ahmad, Imtiaz Ahmed Choudhury, Raja Ariffin Raja Ghazilla

**Affiliations:** Centre for Product Design and Manufacturing (CPDM), Department of Mechanical Engineering, Faculty of Engineering, University of Malaya, Kuala Lumpur 50603, Malaysia; E-Mails: smtaslimreza@gmail.com (S.M.T.R.); imtiaz@um.edu.my (I.A.C.); r_ariffin@um.edu.my (R.A.R.G.)

**Keywords:** electromyography (EMG) sensor, kinematics sensor, accelerometer, exoskeleton, fuzzy controller, lower limbs

## Abstract

Human motion is a daily and rhythmic activity. The exoskeleton concept is a very positive scientific approach for human rehabilitation in case of lower limb impairment. Although the exoskeleton shows potential, it is not yet applied extensively in clinical rehabilitation. In this research, a fuzzy based control algorithm is proposed for lower limb exoskeletons during sit-to-stand and stand-to-sit movements. Surface electromyograms (EMGs) are acquired from the *vastus lateralis* muscle using a wearable EMG sensor. The resultant acceleration angle along the z-axis is determined from a kinematics sensor. Twenty volunteers were chosen to perform the experiments. The whole experiment was accomplished in two phases. In the first phase, acceleration angles and EMG data were acquired from the volunteers during both sit-to-stand and stand-to-sit motions. During sit-to-stand movements, the average acceleration angle at activation was 11° – 48° and the EMG varied from −0.19 mV to +0.19 mV. On the other hand, during stand-to-sit movements, the average acceleration angle was found to be 57.5°–108° at the activation point and the EMG varied from −0.32 mV to +0.32 mV. In the second phase, a fuzzy controller was designed from the experimental data. The controller was tested and validated with both offline and real time data using LabVIEW.

## 1. Introduction

The power exoskeleton is one of the most salutary inventions of modern science. Immense progress in medical science has decreased death rates and as a result, the number of elderly persons has increased. Unfortunately, many of them suffer from neuromuscular disorders, e.g., hemiplegia [1,2], tremors [3,4] *etc*. Exoskeletons are designed to provide support for human movement. The support that is provided by this device is not only useful in human neuromuscular rehabilitation, but can also be exploited to augment the strength of healthy people [5,6].

Research on exoskeletons has been conducted since 1960 [7]. The Berkeley Lower Extremity EXoskeleton (BLEEX) was proposed by researchers at the University of California, Berkeley [8]. They came up with seven degree of freedom (dof) per leg system. The Hybrid Assistive Limb (HAL) calculated virtual torque to assist lower limb movement through surface EMG data [9]. The Active Leg EXoskeleton (ALEX) is able to assist stroke survivors by providing Robotic Assistive Gait Training (RAGT) [10]. Another device, the Cable-driven Arm EXoskeleton (CAREX) has been described in [11]. A gravity balancing exoskeleton is also designed and reported in [12]. Veneman *et al.* have described a LOwer extremity Powered ExoSkeleton (LOPES) which functions as a kinaesthetic interface [13]. Many techniques have also been developed to ensure proper human-robot interaction [14,15]. Another proposed exoskeleton which was able to reduce the metabolic cost significantly is proposed in [16]. Metabolic adaption has been described and reduction of metabolic cost of around 9% has been achieved by Galle *et al.* [17]. Positives outcomes have been found in EMG analysis of the Tibion Bionic Leg (TBL) [18].

Information about human movements can be obtained from the brain, brain stem or spinal cord [19]. Achieving that information from the last terminal *i.e.*, from the muscles, is also an appropriate idea, therefore the electromyography (EMG) signal is considered as the most powerful biological signal to detect human motion intentions [20,21]. Since the surface EMG is contaminated with noise during acquisition, it is important to process that raw EMG signal [22].

Given that the human body is full of signal fuzziness, surface EMG signals are also affected by fuzziness [23]. Consequently, to develop a power assist exoskeleton, an intelligent control system is required. A neuro-fuzzy controller for upper limbs was proposed in [23–26]. A neuro-fuzzy controller for a lower limb exoskeleton has been described in the literature [7]. For lower limbs, a torque controller has been proposed by Christian and Günter [19]. Chan *et al.* described a fuzzy EMG classifier for prosthesis control [27]. They compared their throughput with another Artificial Neural Network (ANN)-based classifier using the same data set as well as the same features. Their findings showed that the fuzzy classifier was superior to the ANN one, because of a higher recognition rate, insensitivity to overtraining, as well as a comparatively consistent throughput.

Hence, a fuzzy based control system demands the investigation of its feasibility for designing a robotic exoskeleton. Per our knowledge, such a type of controller has not been reported yet. In this paper, a fuzzy controller for sit-to-stand and stand-to-sit movements is proposed. Wearable EMG and kinematic sensors are used to accomplish the experiment. An algorithm is developed for data acquisition and filtering of raw EMG signals as well as accelerometer data. Five randomly selected subjects were used to validate the controller employing real time data. The results support the hypothesis which expresses that the developed controller can detect human motion and drive the motor in a necessary direction. Section 2 describes the experimental methodology along with the acceleration angle measurement, EMG signal processing, and a short discussion of the experiments. Section 3 includes a brief description of the design of a fuzzy controller. The results are presented and discussed in Section 4. Finally, conclusions have been drawn by combining all of the important points of the study.

## 2. Experimental Methodology

**Figure 1.**
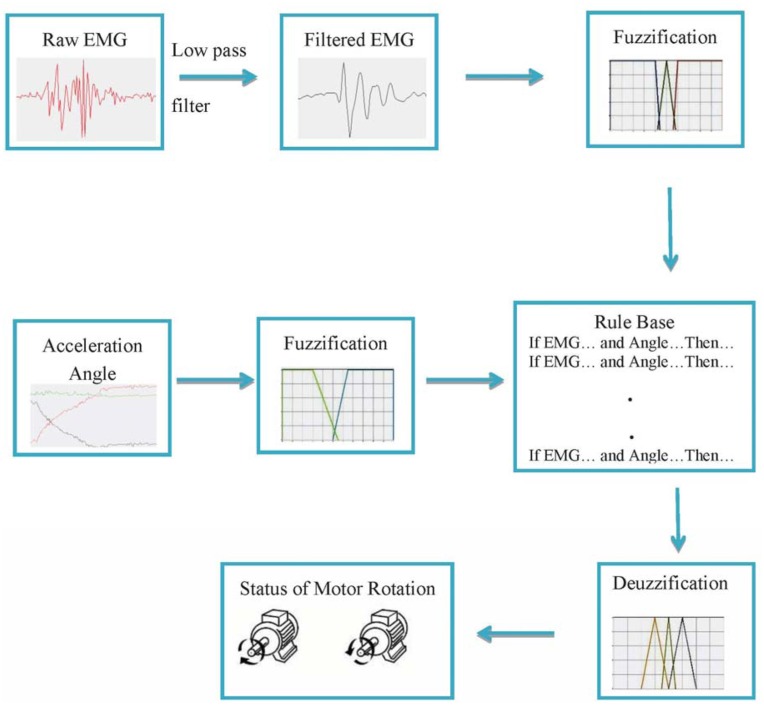
Overall block diagram of the study.

The study has been completed in two phases. In the first phase, acceleration angle and EMG data are collected from the selected subjects. The acceleration angle measurement technique and the EMG data processing are described in this section. In the second phase, a fuzzy controller is designed and validated with the data acquired in the first phase. Figure 1 illustrates the block diagram of whole process.

### 2.1. Acceleration Angle Measurement

**Figure 2.**
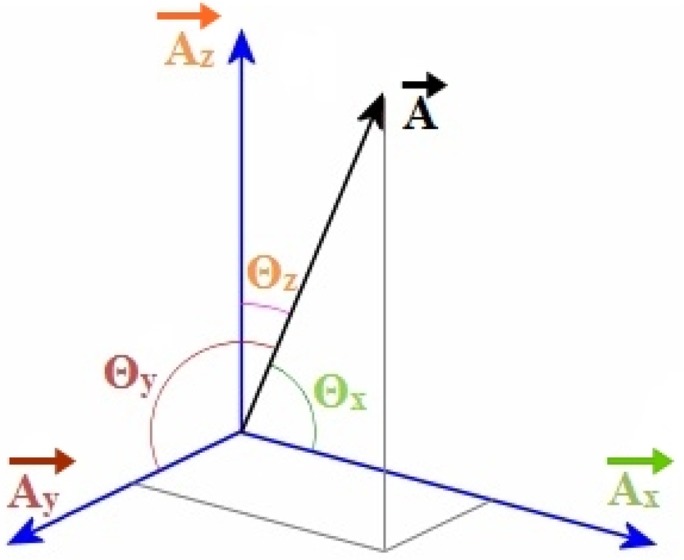
Acceleration components in three axes.

Measuring acceleration angle is the first step to detect the human movement intentions through muscle activation. Three components of acceleration are shown in Figure 2. The direction cosines [28] of the resultant acceleration along three axes are calculated from following equations:

(1) $$\begin{array}{c} \cos\theta_{x}=\frac{A_{x}}{\vec{\left|A\right|}}\\ \theta_{x}=\;\cos^{-1}\left(\frac{A_{x}}{\sqrt{A_{x}^{2}+A_{y}^{2}+A_{z}^{2}}}\right),\;\text{Since},\;\vec{\left|A\right|}\;=\;\sqrt{A_{x}^{2}+A_{y}^{2}+A_{z}^{2}} \end{array}$$

(2) $$\theta_{y}=\;\cos^{-1}\left(\frac{A_{y}}{\sqrt{A_{x}^{2}+A_{y}^{2}+A_{z}^{2}}}\right)$$

(3) $$\theta_{z}=\;\cos^{-1}\left(\frac{A_{z}}{\sqrt{A_{x}^{2}+A_{y}^{2}+A_{z}^{2}}}\right)$$

where, *A_x_*, *A_y_* and *A_z_* are the three components of resultant acceleration *A⃗* along *x*, *y* and *z* axes. θ*_x_*, θ*_y_* and θ*_z_* are the angle between resultant acceleration *A⃗* and the component *A_x_*, *A_y_* and *A_z_* respectively.

**Figure 3.**
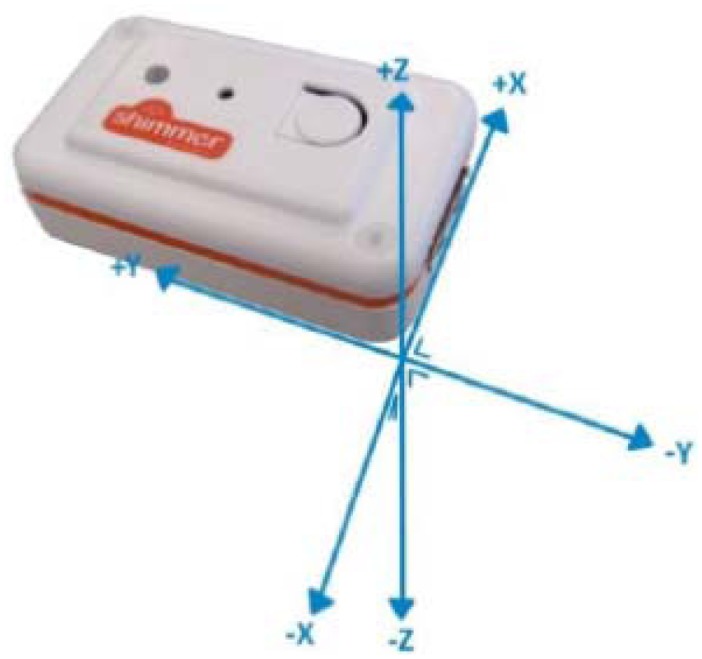
Coordinate system of the kinematics sensor [32].

An accelerometer is a device which measures the acceleration generated because of gravitational and inertial forces as well as profoundly applied in gait analysis [29–31]. A kinematics sensor (Shimmer Technology, Dublin, Ireland) integrated with an accelerometer was used to measure the acceleration. The sensor is 53 mm × 32 mm × 19 mm in dimension and weighs 27 g. The coordinate system of the sensor is illustrated in Figure 3.

**Figure 4.**
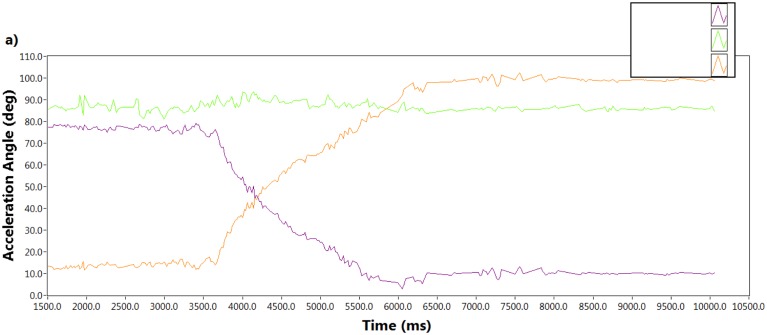

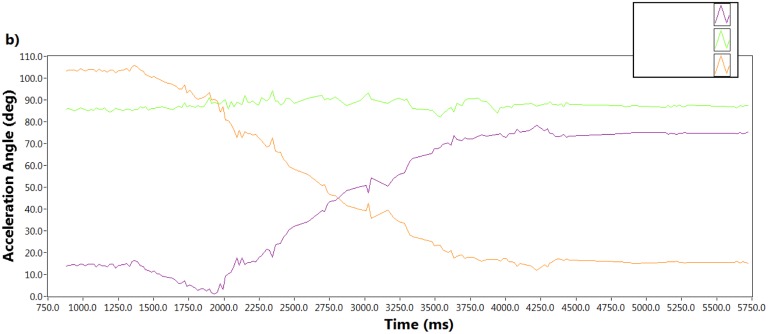
Changes of acceleration angle with respect to time when the sensor rotates around *y*-axis during (**a**) sit-to-stand; (**b**) stand-to-sit.

The sensor was attached on the thigh so that it could be placed on the *xy*-plane. When the person intends to move, *i.e.*, the movement between sit to stand or stand to sit, it was rotated around the *y*-axis. Figure 4a shows the angle calculated from the direction cosines of the resultant acceleration during sit to stand and Figure 4b shows the same during stand to sit along the *x*, *y* and *z* axes with respect to time. The acceleration angle along the *x* axis was decreasing with time whereas the angle along the *z* axis was increasing during sit to stand. On the contrary, during the stand to sit movement the acceleration angle along the *x* axis was increasing and the angle along the *z* axis was decreasing with respect to time. The acceleration angle along the *y*-axis remained the same in both cases.

### 2.2. EMG Signal Processing

EMG is a technique which involves recording and analyzing the electrical activities of muscles at rest and throughout contraction. A wearable EMG sensor (Shimmer Technology) was used to ascertain the muscle activity. The dimensions of the sensor are 53 mm × 32 mm × 23 mm, its weight is 32 g and it is connected to a positive, negative and neutral electrode.

**Figure 5.**
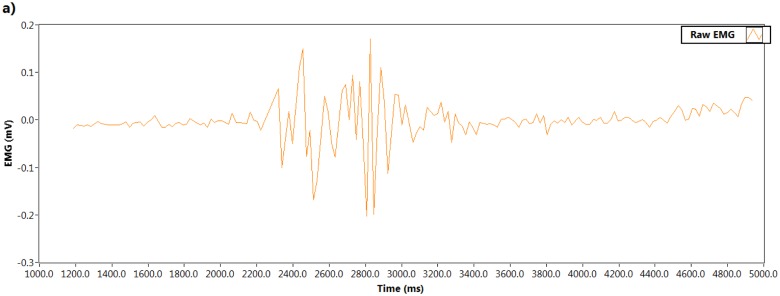

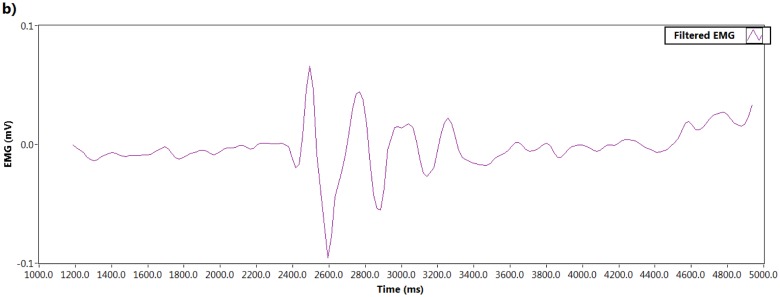
EMG signal processing (**a**) raw EMG; (**b**) filtered EMG.

Naturally raw EMG signals are random in shape due to the constant changes of the actual sets of recruited motor units. EMG signals can be affected by many other issues, e.g., different thickness of tissues, noisy electrical environments, lower grade electrodes, *etc.* that can add noise, but the EMG signal contains very important information about muscle innervations. The noise frequencies that contaminate raw EMG have to be properly filtered out. To remove noise, a Butterworth third order low pass filter was used in this experiment. The cutoff frequency was set at 25 Hz. Figure 5a illustrates the raw EMG signal and Figure 5b shows the filtered signal.

### 2.3. Experiments

In order to identify the lower limb muscle activation with the change of acceleration angle along the *z* axis, twenty volunteers (age 23–30, all male) were picked randomly from a pool of candidates. Participants were asked to complete an informed consent form before the experiment. The consent form included their age, gender, diseases or disabilities, *etc.* Subjects that suffered from any previous neuromuscular injury were excluded from the experiments.

**Figure 6.**
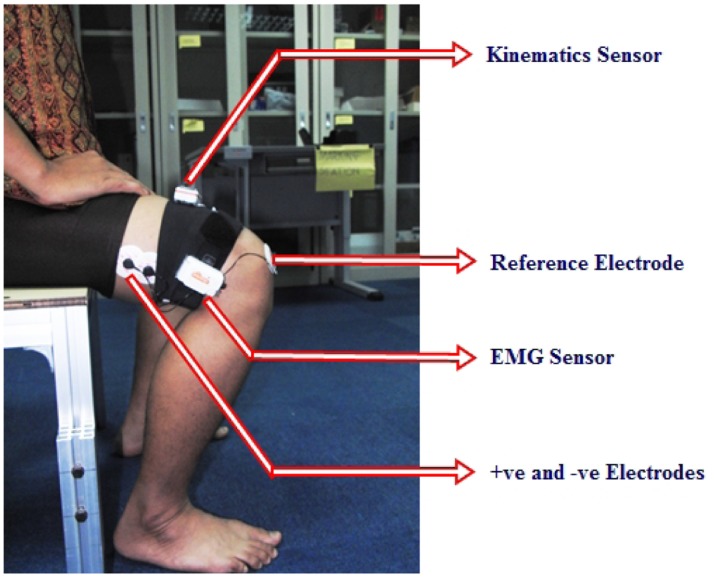
Positioning of the sensors and electrodes.

For the purpose of detection of movement intention, the muscle named *vastus lateralis* was selected as the EMG source. The surface of the muscle was cleaned with sanitizer to remove any inhibitory particles and the hair was shaved to get the best readings. One EMG sensor was placed and bound on the thigh and positive and negative electrodes were positioned on the *vastus lateralis*. The neutral electrode was set at the knee. One kinematics sensor was put on the same thigh so it could rotate around the *y*-axis during sit to stand and stand to sit movements. The positioning of the sensors and electrodes is shown in Figure 6.

The experiments were performed like people's everyday movement when standing up from a chair or sitting down on a chair. Two legs were placed parallel and subjects were asked to get up without holding anything in their arms. They were also encouraged to put same weight in their two feet during the experiments. Volunteers were apprised to perform sit to stand and stand to sit movements as naturally as possible to have to the best results. All movements were performed on the same chair (height 40 cm).

**Figure 7.**
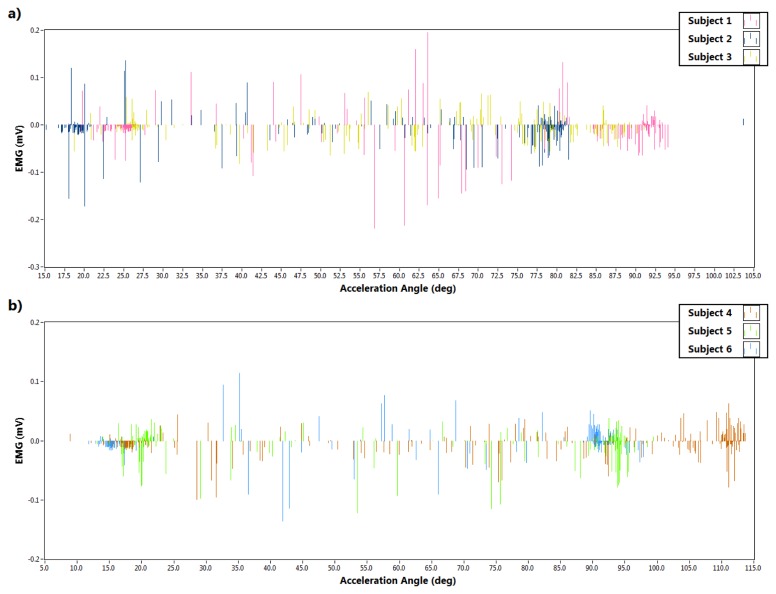

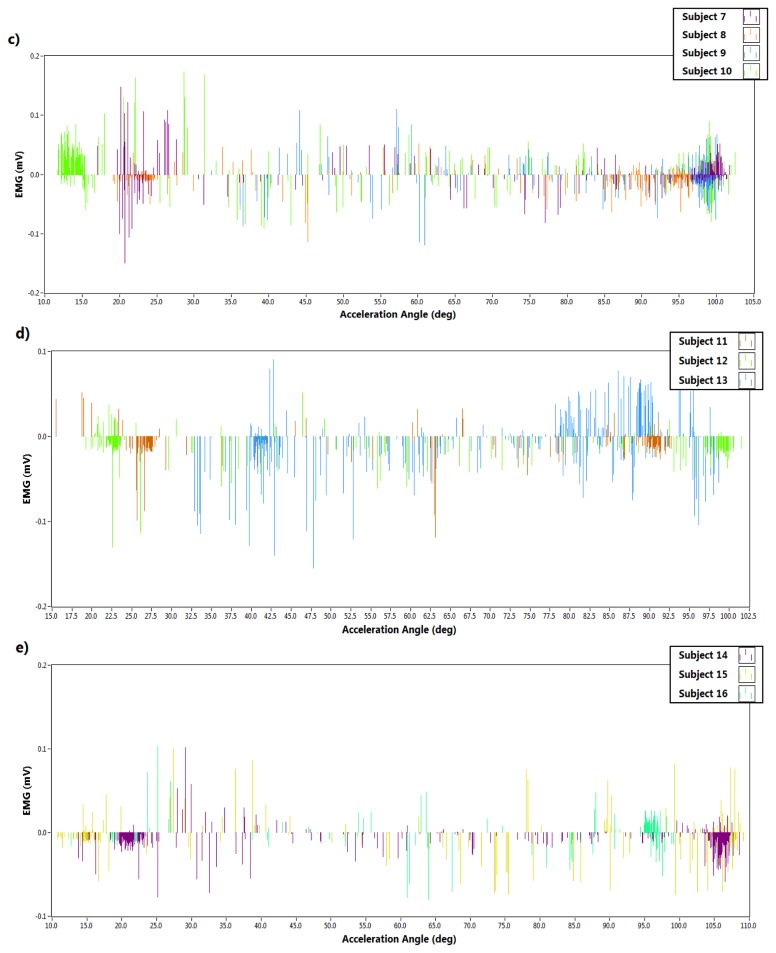

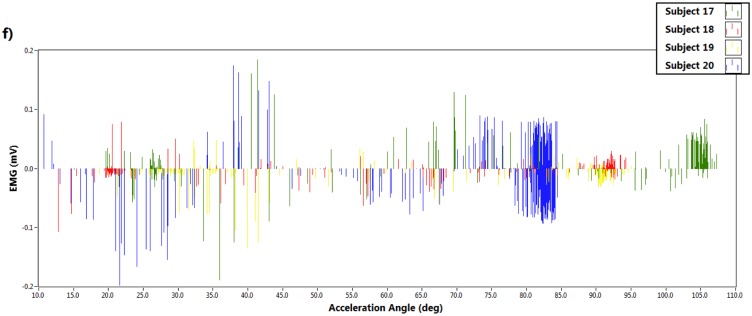
EMG signals of the *vastus lateralis* muscle during sit-to-stand. (**a**) Subjects 1–3; (**b**) Subjects 4–6; (**c**) Subjects 7–10; (**d**) Subjects 11–13; (**e**) Subjects 14–16; (**f**) Subjects 17–20.

**Figure 8.**
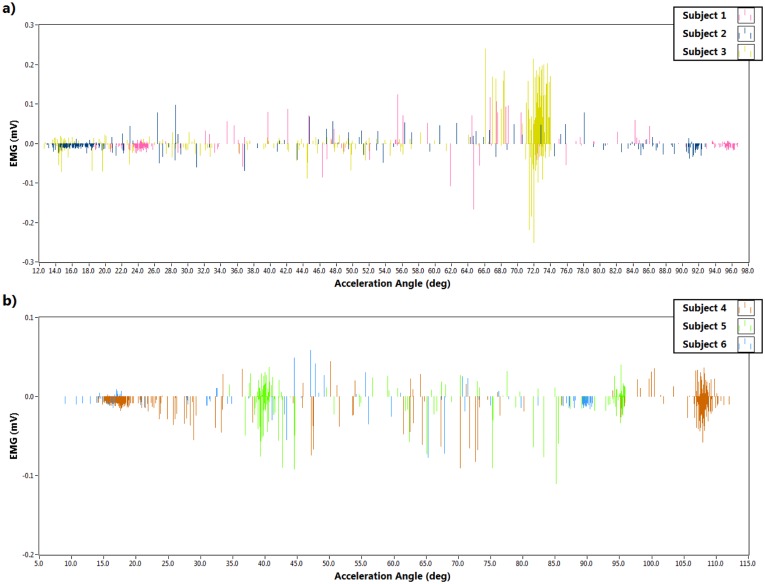

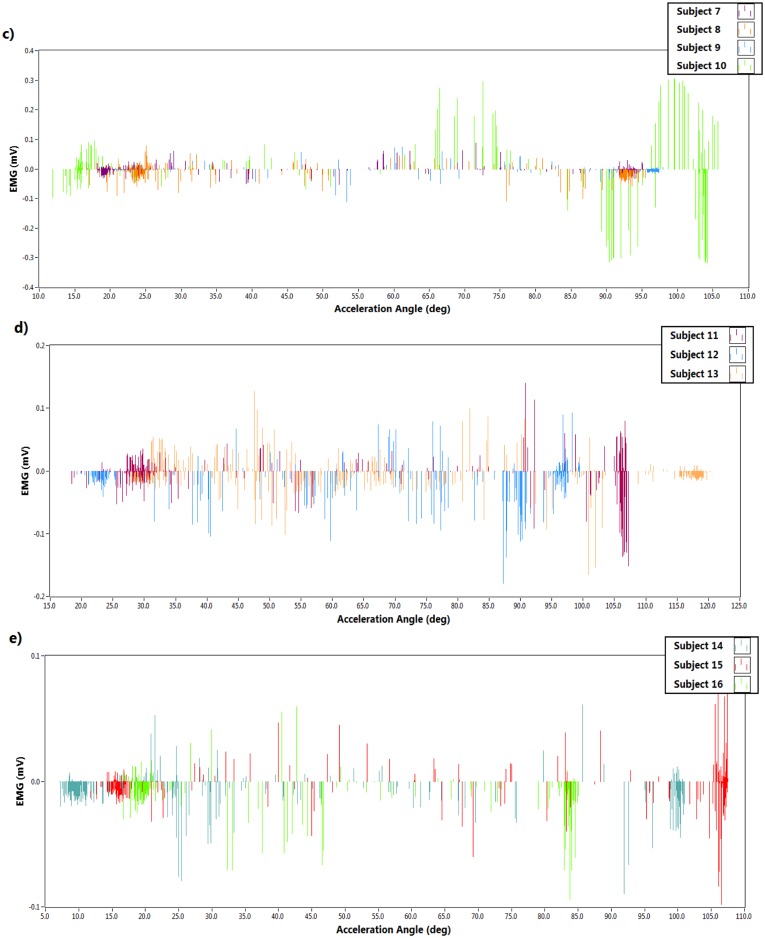

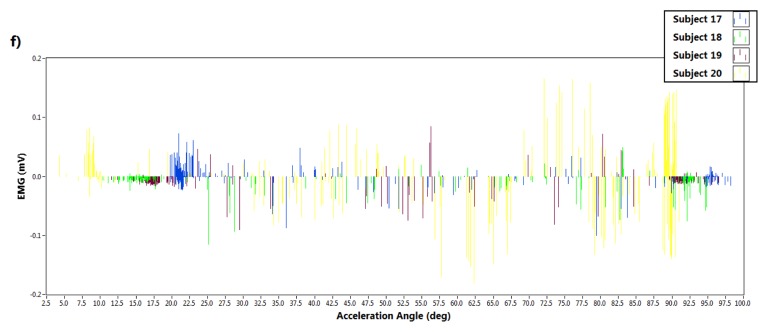
EMG signals of the *vastus lateralis* muscle during stand-to-sit. (**a**) Subjects 1–3; (**b**) Subjects 4–6; (**c**) Subjects 7–10; (**d**) Subjects 11–13; (**e**) Subjects 14–16; (**f**) Subjects 17–20.

Furthermore, data acquisition was performed using LabVIEW (National Instruments, Austin, TX, USA). Only the accelerometer data was extracted from the kinematics sensor over the accelerometer range ±1.5 g. The EMG and the accelerometer data was taken at a 51.2 Hz sampling rate. Figure 7 illustrates the EMG signal during sit-to-stand movement with respect to the resultant acceleration angle along the *z* axis (θ*_z_*) for all participants. Similarly the EMG signal throughout the stand-to-sit movements with the change of the same acceleration angle (θ*_z_*) is depicted in Figure 8. The Graphs were plotted by LabVIEW as well. The findings from the experiments are described in Table 1.

When the subjects intended to stand up or sit down, the range of immediate acceleration angles were selected based on the maximum EMG values. The maximum EMG values in those ranges are presented in Table 1. During sit-to-stand the acceleration angle varied from 11° to 48° and the maximum EMG values were ±0.19 mV, but in the stand-to-sit movement the acceleration angle varied from 57.5° to 108° and maximum EMG was found to be ±0.32 mV. This data was considered when designing the fuzzy controller which is explained in the following section.

## 3. Designing a Fuzzy Controller

A fuzzy controller is an intelligent control system due to having ability of tolerance in membership degree ranging between 0 to 1 where as traditional Boolean logic is either 0 or 1. Fuzzy control was originally proposed by Mamdani and Baaklini [33] and was originally conceptualized by Zadeh in 1965. Being an easy method to express the ambiguous terms of daily life, the fuzzy controller quickly became popular. Eventually a large number of interesting applications have been proposed, e.g., in energy storage [34], in renewable energy [35], in sustainable manufacturing assessment [36], in vehicle control [37–39], in medical science [40], and even in the education sector [41–44]. EMG data is very unpredictable and contains a lot of fuzziness. Therefore, the fuzzy controller is an ideal approach for developing an EMG-based exoskeleton control system.

### 3.1. Defining Linguistic Variables and Membership Function

**Figure 9.**
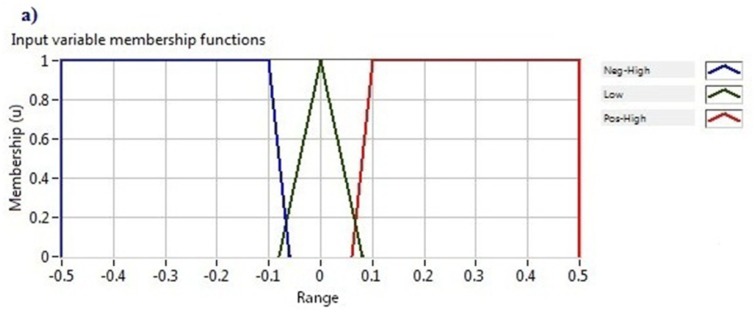

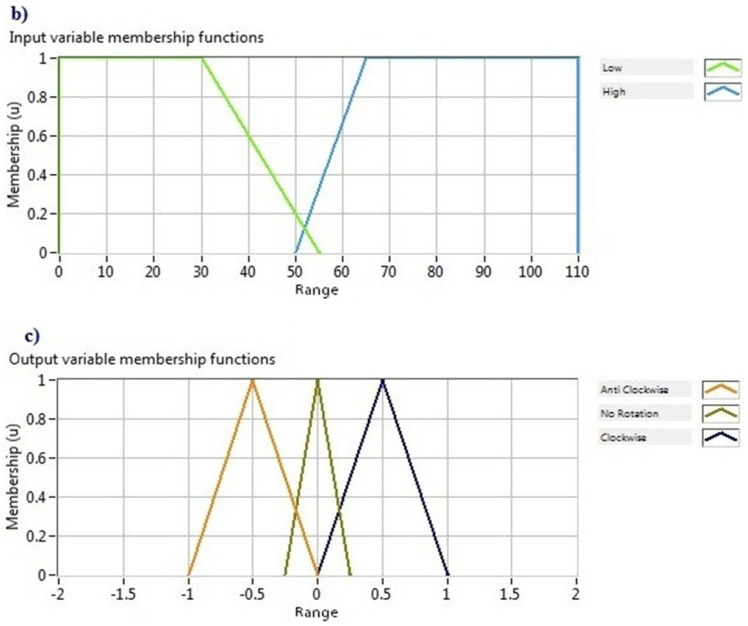
Input and output membership functions (**a**) EMG signal; (**b**) Acceleration angle; (**c**) Motor status.

The proposed controller consisted of two input linguistic variables: EMG signal and acceleration angle and one output linguistic variable: motor status. The linguistic variable EMG signal included the linguistic terms negative high, low and positive high. The other linguistic variable input, the acceleration angle, comprises two linguistic terms named low and high. The output linguistic variable, *i.e.*, motor status, contains anti-clockwise rotation, no rotation and clockwise rotation linguistic terms. The membership functions of those variables were defined from the data that are given in Table 1. The graphical representation of all membership functions is shown in Figure 9.

### 3.2. Defining Fuzzy Rule Base

Fuzzy rules express the relationship between input and output linguistic variables in terms of linguistic terms. The antecedent part, *i.e.*, the If portion and the consequent part, *i.e.*, the Then portion have to be specified as well as the contradictory rules should be avoided due to achieve consistent rule base. If *P*_1_, *P*_2_, *P*_3_ ….. *P_N_* are the linguistic terms of input variables, the total possible rules *N* can be written as:

(4) $$N=P_{1}\times P_{2}\times P_{3}\ldots ..\times P_{N}$$

Therefore, six rules (3 × 2) can be applied in the proposed controller. The rule base in a matrix form is shown in Table 2.

### 3.3. Defuzzification

Defuzzification is a technique that converts the degree of membership of the output variables into a crisp value within their linguistic terms. Several mathematical methods are proposed for defuzzification, such as, center of area (CoA), modified center of area (mCoA), center of sums (CoS), center of maximum (CoM), mean of maximum (MoM) *etc.* To defuzzify the output variable of the proposed fuzzy controller, the modified center of area (mCoA) method was used. This method considers the full area under the scaled membership functions and calculates the geometric center of that area using the following equation:

(5) $$\text{mCoA}=\frac{\int f(x)\text{xdx}}{\int f(x)dx}$$

### 3.4. Testing the Controller

**Figure 10.**
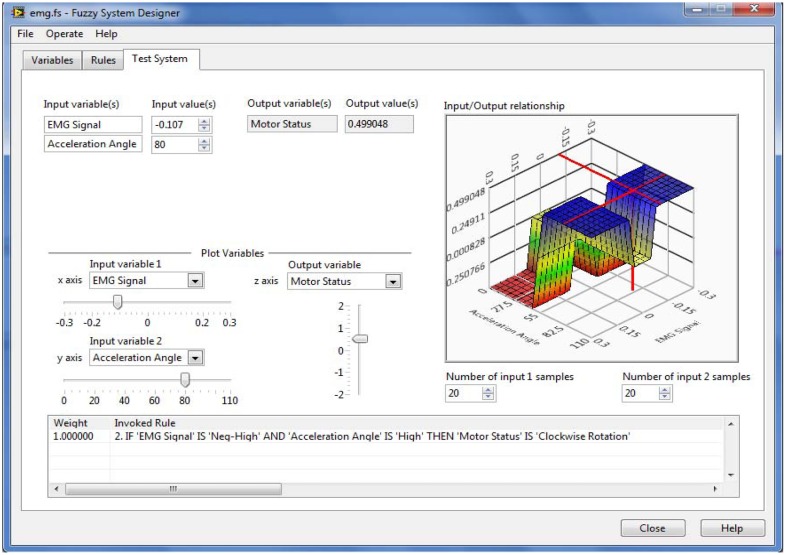
Offline data testing through “Test System”, when “EMG Signal” value is −0.107 and “Acceleration Angle” is 80°, Motor Status is Clockwise Rotation.

The above mentioned fuzzy system was designed in LabVIEW. To validate the rule base, a “Test System” page was used. The input values for two inputs were put randomly and found the appropriate invoked rules. Hence, the system worked deservedly. An example of this test is depicted in Figure 10.

## 4. Results and Discussion

**Figure 11.**
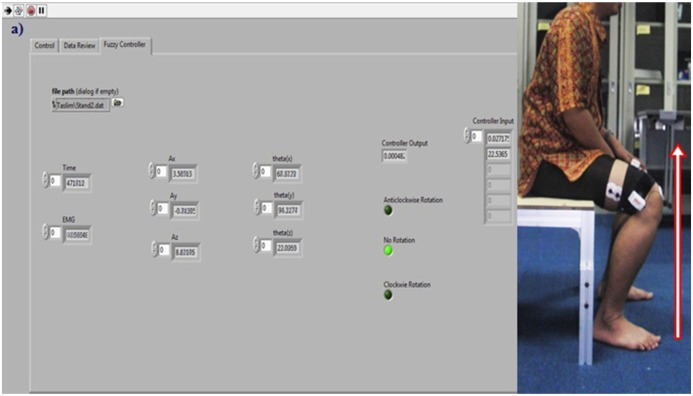

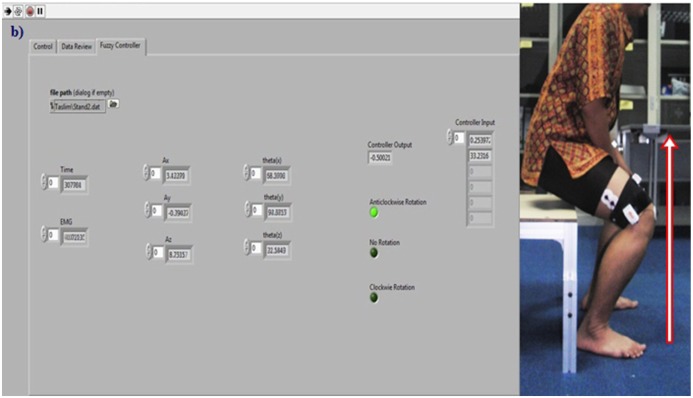
Real time testing of the controller during Sit-to-Stand (**a**) on sit down condition LabVIEW front panel shows the motor status as “No Rotation”; (**b**) when intended to stand up the motor status is changed to “Anti-clockwise Rotation”.

**Figure 12.**
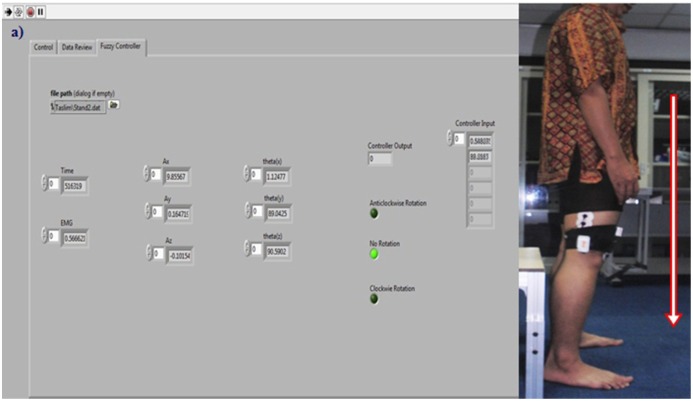

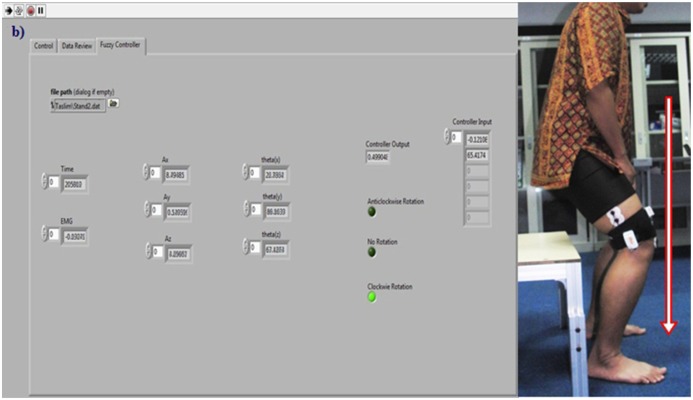
Real time testing of the controller during Stand-to-Sit (**a**) on stand up condition LabVIEW front panel shows motor status as “No Rotation”; (**b**) when intended to sit down the motor status is changed to “Clockwise Rotation”.

The analysis of the EMG signals aimed to investigate how the degree of membership for the controller could be defined. Secondly, after devising the controller, it was important to test the controller with real time data to assess its effectiveness. To corroborate the developed fuzzy controller, five subjects were selected (male gender, height 162, 165, 171, 168 and 169.5 cm and weight 69, 66, 72, 67.5 and 70 kg, respectively). They were asked to perform the same experiment described in Section 2.3. Sensors were placed in the same manner on the same muscle while the same precautions were taken. Figure 11 shows the controller output during sit to stand and Figure 12 illustrates the throughput of the controller during the stand to sit movement of Subject 1. The acceleration angle, EMG value, controller output and the status of motor rotation those were captured during real time tests of five subjects are given at Table 3. In Table 3 the instantaneous values of EMG and acceleration angle as well as their corresponding controller output and motor status during sit-to-stand and stand-to-sit movements of the five randomly selected subjects when the controller is tested in real time are presented.

For Subject 1, during sit-to-stand movement the EMG magnitude is 0.0272 mV and the acceleration angle is 22.53° for an instant which generates a controller output of 0.00048 and causes no motor rotation. This motor status means the subject is still at sitting down condition.

On the contrary, when the subject intends to stand up, the EMG magnitude increased to 0.254 mV and the acceleration angle became 33.23°, which produces a controller output of −0.50023 and changed the motor rotation to “Anticlockwise Rotation”. Likewise for the same subject during stand-to-sit movement, the EMG magnitude and acceleration angle for an instant are 0.0483 mV and 88.88°, respectively, which make controller output 0.00000 and motor status remains at “No Rotation”. This status indicates that the subject is standing up. When the subject intends to sit down the EMG magnitudes and acceleration angle change simultaneously to −0.121 mV and 65.42°, respectively. These changes reset the controller output to 0.49990 and the motor status to “Clockwise Rotation”. Similar patterns are found for the other subjects as well.

The experimental results show that the designed controller performed properly for the five subjects, thus the proposed controller is capable of detecting the human intention through surface EMG signals. By employing the relation between EMG and acceleration angle it can control the motor direction as required. EMG-based controllers for the upper or lower limb exoskeletons have been widely investigated for the last few decades. Each researcher has come up with his or her own intelligent control system, e.g., fuzzy, ANN, neuro-fuzzy controller, *etc.* This study emphasized the fuzzy controller for a sit to stand and stand to sit movement. Yin *et al.* [7] proposed another human machine interface (HMI) neuro-fuzzy controller for lower limbs. They established an extended physiological proprioception (EPP) feedback system for lower limb exoskeletons, but it was not validated for sitting and standing movements.

On the contrary, Christian and Günter [19] evaluated their developed exoskeleton for sit-to-stand movements, but the control algorithm was based on torque control which was estimated from the EMG signals. Another important feature was the use of six channels to acquire EMG signals whereas the proposed fuzzy controller uses only one channel, hence a much simplified controller is developed in this research.

## 5. Conclusions

In this paper, a fuzzy controller for lower limb exoskeletons has been presented. In order to ensure the simplicity of the hardware, wearable sensors were used. The EMG data acquisition is completed through a single channel which makes the controller handier. In the first phase of the experiment acceleration angles and EMG data were collected from twenty volunteers. The acceleration angles varied from 11° to 48° during sit-to-stand movements and the maximum EMG is ±0.19 mV whereas during stand-to-sit movements the acceleration angle varied from 57.5° to 108° and the maximum EMG is found to be ±0.32 mV. Using this data, the fuzzy controller is proposed and tested with offline data.

The developed fuzzy controller is also tested using real time data. Five randomly selected subjects were used in the test and the experimental results show the power of the controller. The controller output for sit-to-stand from the real subjects falls within the range of either 11° to 48° acceleration angles or EMG values ±0.19 mV. In the same way during stand-to-sit movement the controller output for the same subjects falls within the range of either a 57.5° to 108° acceleration angle or EMG values ±0.32 mV. This study can be considered as the initial part of building a prototype exoskeleton for lower limb impairment.

In order to have a precise and stable control, some other EMG signal processing technique, e.g., root mean square (RMS), moving average (MAV), or wavelet analysis, *etc.* need to be introduced. Future work will aim to develop the controller to increase the degree of freedom (dof) range of human movement as well as to be compatible with robust application.

## Figures and Tables

**Table 1.** Range of acceleration angle and corresponding EMG values during sit-to-stand and stand-to-sit movements.

**Volunteers**	**Height (cm)**	**Weight (kg)**	**Sit to stand**		**Stand to sit**	

			**Acceleration angle during activation**	**Approximate EMG at activation (mV)**	**Acceleration angle during activation**	**Approximate EMG at activation (mV)**
**Subject 1**	162.5	68	18°–25°	±0.08	65°–69°	±0.12
**Subject 2**	167	71	16°–25°	±0.15	73°–79°	+0.1
**Subject 3**	165	76	26°–40°	±0.07	66°–75°	±0.2
**Subject 4**	167	70	27°–32°	−0.1	64°–75°	−0.08
**Subject 5**	166	72	20°–53°	−0.1	75°–86°	−0.1
**Subject 6**	165	69	32°–43°	±0.12	65°–68°	−0.07
**Subject 7**	160	67	18°–21°	±0.15	70°–73°	+0.1
**Subject 8**	162.5	70	33°–45°	−0.12	76°–87°	−0.12
**Subject 9**	175	74	37°–45°	+0.1	60°–68°	±0.08
**Subject 10**	165	71	21°–32°	+0.17	90°–108°	±0.32
**Subject 11**	168	69	16°–27°	−0.1	90°–107°	±0.15
**Subject 12**	171	73	22°–26°	−0.13	87°–100°	−0.19
**Subject 13**	167.5	71	32°–48°	−0.16	81°–103°	−0.18
**Subject 14**	166	68	17°–38°	+0.1	87°–102°	−0.09
**Subject 15**	169	67.5	14°–40°	+0.1	105°–108°	±0.1
**Subject 16**	172	76	24°–28°	+0.1	83°–86°	−0.09
**Subject 17**	171	70	32°–43°	±0.18	79°–85°	−0.09
**Subject 18**	167	69	13°–22°	−0.1	77.5°–95°	−0.08
**Subject 19**	166.5	68.5	30°–43°	−0.12	74°–85°	±0.08
**Subject 20**	170	72	11°–43°	±0.19	57.5°–92°	±0.19

**Table 2.** The rule base of the fuzzy controller.

		**EMG Signal**		

		**Negative High**	**Low**	**Positive High**
**Acceleration Angle**	Low	Anti-clockwise Rotation	No Rotation	Anti-clockwise Rotation
	High	Clockwise Rotation	No Rotation	Clockwise Rotation

**Table 3.** Throughput of the real time tests.

**Subjects**	**Sit-to-Stand**				**Stand-to-Sit**			

	**EMG (mV)**	**Acceleration Angle (deg)**	**Controller Output**	**Motor Status**	**EMG (mV)**	**Acceleration Angle (deg)**	**Controller Output**	**Motor Status**
**Subject 1**	0.0272	22.53	0.00048	NR	0.0483	88.88	0.00000	NR
	0.254	33.23	−0.50023	ACR	−0.121	65.42	0.49990	CR
**Subject 2**	−0.005	14.8	0.00199	NR	0.0232	79.84	0.00093	NR
	0.1100	40.31	-0.4993	ACR	0.1720	83.75	0.48967	CR
**Subject 3**	−0.016	28.48	0.00168	NR	0.0088	103.72	0.00199	NR
	−0.104	26.23	−0.50013	ACR	0.1036	74.59	0.49904	CR
**Subject 4**	−0.003	20.27	0.00199	NR	0.0038	104.607	0.00199	NR
	−0.207	24.86	−0.50013	ACR	0.0947	73.31	0.49872	CR
**Subject 5**	0.0198	5.70	0.00131	NR	-0.056	100.76	0.00569	NR
	0.1592	48.62	0.49673	ACR	0.1052	76.97	0.49904	CR

NB: NR, ACR and CR stand for ‘No Rotation’, ‘Anti-clockwise Rotation’ and ‘Clockwise Rotation’ respectively.
